# Causal relationship between genetically predicted type 2 diabetes mellitus and male infertility

**DOI:** 10.3389/fendo.2024.1357279

**Published:** 2024-03-11

**Authors:** Cuihua Fan, Jiandong Zhang, Dongbiao Qiu

**Affiliations:** ^1^ Department of Blood Transfusion, the First Affiliated Hospital of Fujian Medical University, Fuzhou, Fujian, China; ^2^ Department of Blood Transfusion, National Regional Medical Center, Binhai Campus of the First Affiliated Hospital, Fujian Medical University, Fuzhou, Fujian, China; ^3^ The Center of Information, the First Affiliated Hospital of Fujian Medical University, Fuzhou, Fujian, China; ^4^ The Center of Information, National Regional Medical Center, Binhai Campus of the First Affiliated Hospital, Fujian Medical University, Fuzhou, Fujian, China

**Keywords:** T2DM, male infertility, causal relationship, Mendelian randomization, GWAS

## Abstract

**Background:**

Diabetes mellitus (DM) stands as the most prevalent endocrine abnormality affecting the physiological systems and organs and impairing the male reproductive functions. Type 2 Diabetes Mellitus (T2DM), accounting for about 90-95% of DM, is closely associated with male infertility. However, the magnitude of the causal relationships between T2DM and male infertility remains unclear. The current investigation was to explore the causal relationship between T2DM and male infertility utilizing the Mendelian Randomization (MR) analysis.

**Methods:**

A two-sample MR (2SMR) analysis was conducted to investigate the causal relationship between T2DM and male infertility in the European population from the genome-wide association study (GWAS) summary data that was publicly accessible. GWAS for T2DM and male infertility were extracted from the IEU Open GWAS Project database, with the resulting data encompassing 680 cases and 72,799 controls as the outcome data. Five MR methods were employed for the 2SMR analyses, namely the MR-Egger, weighted median estimation (WME), weighted mode (WM), inverse-variance weighted (IVW), and simple mode. The primary analytical technique utilized in this study was the IVW method, and a multivariate MR analysis was executed to examine the potential mediating influences of T2DM on male infertility.

**Results:**

Following were the odds ratios (ORs) and associated 95% CIs derived from IVW (fixed effects), MR-Egger, WM, WME, and simple mode approaches: 0.824 (95% CI 0.703-0.966), 0.726 (95% CI 0.527-1.001), 0.827 (95% CI 0.596-1.150), 0.841 (95% CI 0.654-1.082), and 0.875 (95% CI 0.544-1.405), respectively. The outcomes of the heterogeneity tests were *P*=0.378 and *P*=0.384, respectively, implying no heterogeneity. Egger-intercept outcomes were *P*=0.374, highlighting the absence of pleiotropy. The stability of the results was affirmed through the leave-one-out analysis. Notably, all *F-*values surpassed 10, indicating the absence of weak bias attributed to instrument variables(IVs).

**Conclusions:**

This research furnishes evidence supporting a causal association between T2DM and male infertility. These insights offer a foundation for future investigations aiming to establish the association between genetically predicted T2DM and male infertility. These outcomes suggest the significance of active monitoring and proactive measures for preventing infertility in male individuals with T2DM. Furthermore, careful consideration is required for individuals of reproductive age to prevent and treat T2DM.

## 1 Introduction

Diabetes mellitus (DM) has emerged as a significant global health challenge marked by elevated morbidity and mortality rates. The prevalence of DM is steadily rising, impacting a growing number of young and middle-aged males. Presently, DM affects approximately 422 million individuals globally and is reported to contribute to 1.5 million fatalities annually (1, 2). DM has reached epidemic proportions in the past. The prevalence of DM has witnessed a substantial escalation, escalating from 108 million cases in 1980 to 537 million cases by the year 2021. Predictions suggest a further increase, estimating that 643 million individuals will have DM by 2030 and 783 million will develop this disease by 2045 (3). Diabetes can be classified into four types based on etiology and pathology, including type 1 diabetes mellitus (T1DM; approximately 5-10% of the DM cases), type 2 diabetes mellitus (T2DM; approximately 90-95% of the DM cases), “other” causes of DM (<5% of the DM cases), and gestational diabetes mellitus (GDM). Recent estimates indicate that the prevalence of DM in the general population of the United States is 9.7%, with T2DM and T1DM constituting 8.5% and 0.5% of the cases, respectively (3). DM, particularly T2DM, exhibits a higher prevalence among males compared to females (4). T2DM exerts a considerable impact on numerous physiological systems and tissues, including the male reproductive organs. As the age of individuals diagnosed with T2DM has been progressively decreasing in recent years, an escalating number of young and middle-aged males are struggling with this disease during their reproductive years.

According to the World Health Organization (WHO), infertility has progressively evolved into a public health concern, presenting a prevalence of around 10%-15%. Notably, the male factor contributes to approximately 40% of infertility cases (5), impacting around one in ten couples within the reproductive age range (6). Declining world fertility rates may have a serious negative impact on social development. Predictions suggest that the worldwide population is expected to reach around 9.7 billion by the year 2064. Nevertheless, it will decrease to approximately 8.8 billion in 2100 (7). Pure or mixed male factor has been recognized in approximately half of the infertility cases, with estimates indicating that up to 12% of males experience fertility issues (6, 8). The etiology of male infertility may be associated with congenital or acquired conditions, spanning a range of factors encompassing pretesticular, testicular, or post-testicular causes (6, 8). Among the different causes of infertility, DM, severe ejaculatory disorders, and erectile dysfunction were considered pretesticular causes of infertility (6). The prevalence of DM in infertile males is estimated to be 0.7%-1.4%. However, some studies have reported that the prevalence of infertility in males with DM ranges from approximately 35%-51%. DM in males appears to exert a discernible adverse impact on the fertility of couples, wherein the status of being childless or subfertile in males may be associated with an elevated risk of developing DM (9). Recent studies have brought to light a substantial correlation between DM and sexual dysfunctions (3, 10), specifically, erectile (3, 11) and ejaculatory dysfunctions (12) as well as hypogonadism (11). Available data underscores the evident roles that DM plays in impairing male reproductive organs, thereby influencing overall couple fertility (9, 13). Therefore, it is well known that the low fertility rate of DM patients in humans.

T2DM is the main type of DM cases. An extensive body of research, spanning both clinical observations and studies involving animals, has dedicated attention to exploring and elucidating the impact of T2DM on various aspects of sperm quality and its associated parameters (10, 14). Generally, the pathophysiological mechanisms of becoming infertile in T2DM is caused by an inflammatory condition with increased oxidative stress resulting in decreased sperm vitality and increased sperm DNA fragmentation (10). There is evidence that prevalence of younger patients with T2DM is estimated at 31% in 10-19 years (15). Nonetheless, findings from a Mendelian randomization (MR) study suggest that the elevated risk of spermatozoa abnormality in male Europeans may not be explained solely by T2DM (16). Subsequent studies necessitate larger sample sizes to elucidate the relationship and potential underlying mechanisms between T2DM and male infertility.

Several studies, encompassing both clinical observations and animal research, have substantiated the relationship between T2DM and male infertility (10, 14, 17). Nonetheless, the conclusion drawn from these studies regarding the causal relationship between T2DM and male infertility exhibited inconsistencies. The identified correlation risk in their findings fell short of offering a comprehensive account, leaving gaps in addressing potential confounding factors, such as socioeconomic status and diverse lifestyles. Consequently, an MR study was executed to examine the possible underlying causal relationship between T2DM and male infertility. MR analysis has emerged as a widely adopted tool for assessing the causal relationship between risk factors and outcomes. This sophisticated analytical approach capitalizes on genetic variants stemming from meiosis, effectively leveraging them as a natural experiment (18–20). Considering the random distribution of genetic variants at conception, MR analysis is less prone to bias from potential reverse causality and confounding compared to observational studies (21, 22).

In cases where data on exposure and outcome are measured in separate samples, an MR study can estimate causal effects through a two-sample MR (2SMR) approach (23). Due to the random classification of genotypes at conception, confounding and bias in 2SMR are limited (24). Hence, a 2SMR analysis was performed in this investigation to examine the causal association between T2DM as the exposure and male infertility as the outcome utilizing summary datasets from the genome-wide association studies (GWAS).

## 2 Methodology

### 2.1 Data sources

T2DM and male infertility data were retrieved from the IEU Open GWAS Project database (https://gwas.mrcieu.ac.uk/) to identify the most relevant GWAS summary data. Individuals of European ancestry were specifically selected for the cohort to mitigate potential errors arising from stratification effects related to factors like ancestry and population. Furthermore, in this study, a preference was given to GWAS data with a larger sample size, encompassing more single nucleotide polymorphisms (SNPs). The study selected SNPs associated with T2DM in Europeans from a GWAS analysis (GWAS-ID: finn-b-E4_DM2), comprising 32,469 patients with T2DM and 183,185 healthy controls of European ancestry (**Table 1**). In this research, genetically and statistically plausible SNPs meeting a genome-wide significance threshold of *P* < 5e-08 were selected. In this context, the genetic variants exhibited a strong association with T2DM in our 2SMR analysis. The *F*-statistic was utilized to examine the potential for weak instrumental bias and the statistical power of individual SNPs. To ensure the exclusion of weak instrument bias, an *F*-statistic cutoff value of *F* < 10 was applied (25). Genetic association data for male infertility in individuals of European ancestry were sourced from the IEU Open GWAS Project database. This dataset comprised a total of 680 individuals with male infertility and 72,799 country-matched non-DM participants of European ancestry (**Table 1**). This study determined the β coefficients and standard errors for overall male infertility associated with each retrieved SNP of T2DM from the GWAS summary statistics in the European population.

**Table 1:** T2DM SNPs used to construct the instrument variable in Europeans.

Chr	Position	SNP	EA	OA	EAF	Beta	SE	P value
2	43453721	rs112694524	A	G	0.0334	-0.1734	0.0294	3.46E-09
2	227121918	rs2943656	G	A	0.6174	0.0752	0.0107	1.72E-12
2	43480221	rs62137406	T	C	0.0490	0.1422	0.0239	2.57E-09
2	59314086	rs139640586	C	A	0.4088	-0.0578	0.0106	4.24E-08
2	60553519	rs17039732	A	T	0.0476	0.1481	0.0243	1.16E-09
2	165508389	rs10184004	T	C	0.3576	-0.0668	0.0108	7.31E-10
3	170629884	rs6786846	A	G	0.6810	0.0654	0.0111	4.18E-09
3	12336507	rs11709077	A	G	0.1706	-0.1087	0.0138	3.69E-15
3	186665645	rs3887925	T	C	0.4630	0.0591	0.0104	1.39E-08
3	23407658	rs6550758	C	A	0.8216	-0.0826	0.0135	1.05E-09
3	123124513	rs71330995	A	G	0.1878	-0.0860	0.0133	1.10E-10
3	185503456	rs6780171	A	T	0.3066	0.0929	0.0112	1.22E-16
4	45182527	rs10938397	G	A	0.4736	0.0682	0.0104	5.20E-11
4	6315406	rs13143143	G	A	0.5422	0.0789	0.0104	3.98E-14
5	102143311	rs76177300	A	G	0.0578	0.1409	0.0222	2.32E-10
6	140291319	rs1933742	T	G	0.1913	-0.0774	0.0132	4.90E-09
6	7245458	rs1815311	G	A	0.4053	0.0620	0.0106	5.21E-09
6	20680678	rs9348441	A	T	0.3289	0.1230	0.0110	4.91E-29
6	32710407	rs115018313	C	T	0.0503	0.3246	0.0240	1.01E-41
6	32789739	rs73410774	T	G	0.0523	0.3049	0.0234	1.04E-38
6	32932620	rs112511187	AAAACAAACAAAC	A	0.0477	0.3143	0.0248	8.72E-37
7	102086552	rs77655131	T	C	0.1835	0.0968	0.0134	6.08E-13
7	44255643	rs878521	A	G	0.2071	0.0784	0.0128	8.94E-10
7	28256240	rs498475	A	G	0.6448	-0.0625	0.0108	8.27E-09
7	150537635	rs62492368	A	G	0.3402	0.0740	0.0110	1.52E-11
8	118185733	rs11558471	G	A	0.3784	-0.0753	0.0107	1.90E-12
9	22137685	rs7018475	G	T	0.2790	0.1126	0.0116	2.53E-22
9	22132698	rs10965246	C	T	0.1518	-0.1276	0.0145	1.64E-18
9	139248082	rs28642213	G	A	0.6974	0.1005	0.0113	6.25E-19
9	4291928	rs10974438	C	A	0.3757	0.0584	0.0107	4.90E-08
10	71449878	rs182788819	T	C	0.0387	0.1488	0.0269	3.16E-08
10	12309268	rs11257658	A	G	0.2648	0.0827	0.0118	2.70E-12
10	114754071	rs34872471	C	T	0.2016	0.2962	0.0131	1.17E-112
10	94460650	rs10882099	C	T	0.4769	-0.0818	0.0104	3.21E-15
10	114737633	rs144155527	T	C	0.0236	-0.2116	0.0345	8.87E-10
11	2858546	rs2237897	T	C	0.0814	-0.1782	0.0192	2.08E-20
11	72463435	rs7109575	A	G	0.2374	-0.0927	0.0122	3.88E-14
11	92708710	rs10830963	G	C	0.3567	0.1183	0.0108	8.92E-28
11	17408630	rs5215	T	C	0.5284	-0.0586	0.0104	1.62E-08
12	4365572	rs74862545	T	C	0.0195	-0.2531	0.0388	6.88E-11
12	32690857	rs4931017	G	A	0.6926	0.0625	0.0113	3.07E-08
12	66170481	rs2583921	C	A	0.0563	0.1526	0.0226	1.45E-11
12	71526677	rs1397566	G	A	0.4275	-0.0596	0.0105	1.25E-08
12	121432117	rs56348580	C	G	0.2827	-0.0786	0.0116	1.25E-11
12	4384844	rs76895963	G	T	0.0312	-0.4842	0.0325	2.93E-50
12	4521511	rs78470967	A	T	0.0394	-0.2295	0.0273	4.33E-17
12	4271088	rs112108223	A	G	0.0224	-0.3623	0.0365	2.89E-23
13	80718654	rs7998259	A	G	0.3894	-0.0727	0.0107	1.07E-11
15	77892857	rs58102377	G	A	0.4094	-0.0600	0.0106	1.41E-08
16	75236763	rs55993634	G	C	0.0872	-0.1449	0.0185	5.60E-15
16	77261943	rs12449219	G	C	0.0584	0.1342	0.0223	1.62E-09
16	53818167	rs9933509	C	T	0.4129	0.1161	0.0105	2.62E-28
17	36103565	rs11263763	A	G	0.6457	-0.0684	0.0109	3.05E-10
17	36047417	rs3110641	G	A	0.7867	0.0715	0.0128	2.51E-08
18	57908675	rs11665052	G	A	0.1947	0.0775	0.0131	3.00E-09
19	19393714	rs8100204	A	G	0.1598	0.0956	0.0143	2.11E-11
19	7976529	rs2303700	C	T	0.6741	-0.0649	0.0111	5.08E-09
19	45411941	rs429358	C	T	0.1825	-0.0821	0.0136	1.63E-09
19	46157004	rs10408179	C	T	0.4465	-0.0593	0.0105	1.39E-08
20	57607363	rs45551238	T	C	0.0499	-0.2345	0.0243	5.50E-22
20	42888082	rs6073386	G	A	0.0367	0.1790	0.0273	5.93E-11
22	20796117	rs8353	T	G	0.3009	-0.0686	0.0113	1.34E-09

Chr, chromosome; SNP, single nucleotide polymorphism; EA, Effect Allele; OA, Other Allele; EAF, effect allele frequency; SE, standard error.

### 2.2 Instrumental variables

**Figure 1:**
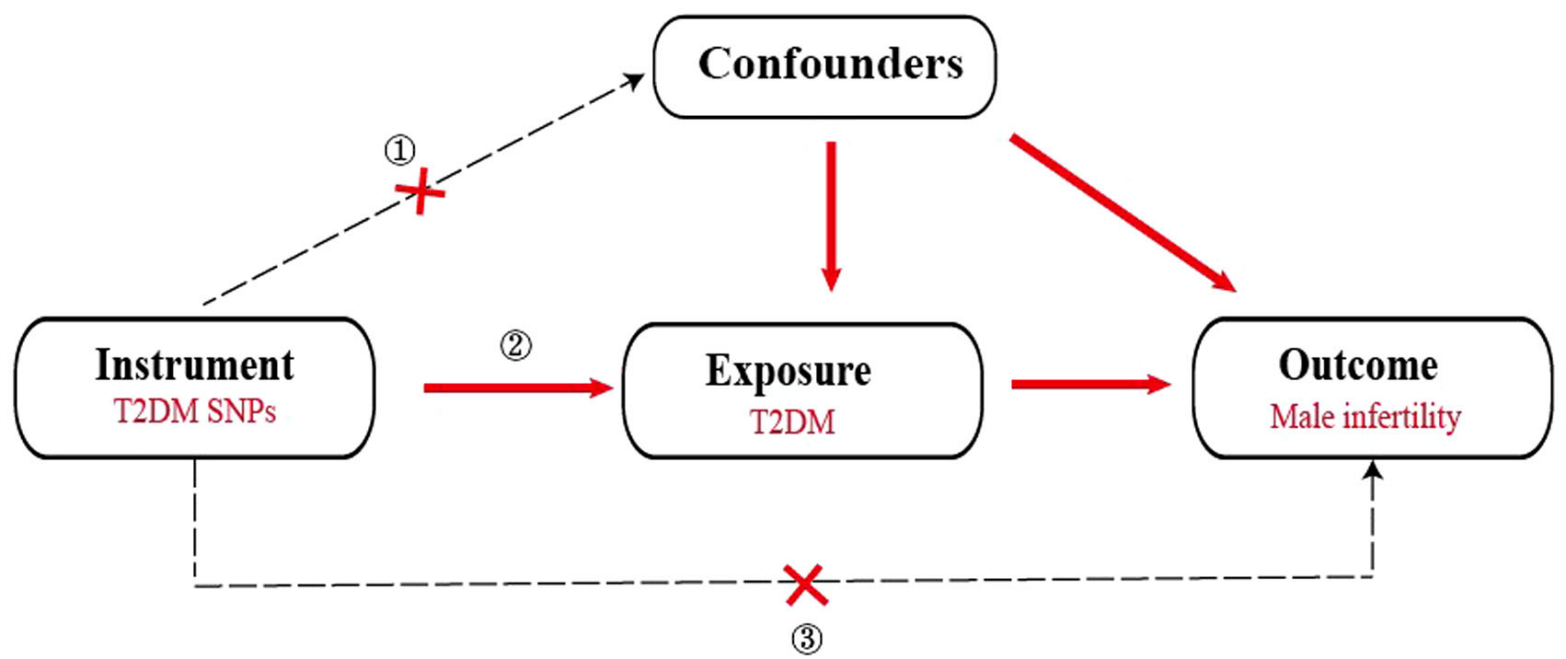
Overall design of the two-sample Mendelian randomization analysis in this study.

To ensure the effectiveness of instrumental variables (IVs), the SNPs used as IVs in the 2SMR analysis must satisfy the subsequent conditions (1): demonstrate a strong association with T2DM (2), exhibit no association with any confounding factors related to both T2DM and male infertility and (3) affect male infertility solely through the pathway of T2DM (**Figure 1**). Hence, the selection criteria to identify the effective SNPs were as follows (1): SNPs chosen as potential IVs were linked to each genus at the locus-wide significance threshold (*P <*5×10^-8^) (2). The linkage disequilibrium (LD) between the SNPs was calculated utilizing 1000 Genomes Project data for the European population as the reference panel. Out of these, SNPs exhibiting an *r* ^2^<0.001 (clumping window size=10,000 kb) were retained, including only those with the most minimal *P*-values (3). SNPs with minor allele frequency (MAF) ≤0.01 were excluded, and in cases where palindromic SNPs were identified, the inferring of forward strand alleles was made utilizing allele frequency information.

### 2.3 Statistical analysis

#### 2.3.1 Linkage disequilibrium assessment

Genetic variants employed as IVs in most MR methods must be independent of confounding factors, prohibiting the existence of LD. According to the hypothesis, the correlation LD between the chosen SNPs and potential confounding factors was assessed.

#### 2.3.2 Main MR analysis

In our MR study, five approaches employing a multiplicative random effects model were utilized. These methods include Mendelian randomization-Egger regression (MR-Egger), weighted median estimation (WME), inverse-variance weighted (IVW), weighted mode (WM), and simple mode. Notably, the random effects IVW method stands out as the most extensively employed and accepted approach in MR analysis. This is attributed to its capability to consider a large number of SNPs and address the substantive observed heterogeneity during the analysis of causality (26, 27). The MR-Egger method offers a robust measure of causal effects that adjust for horizontal pleiotropy. This is achieved by pooling a single SNP-specific Wald ratio utilizing adaptive Egger regression (28, 29). The WME method offers a consistent estimate of causal effects by utilizing the weighted median of Wald under the condition that at least 50% of variants adhere to the criteria of a valid IV for the exclusion restrictions. Utilizing the estimation of individual proportions, the WM method categorizes SNPs based on their similarity and computes the counter-variance weighted count of SNPs in each group. Ultimately, it derives a causal estimate according to the group of SNPs by the largest weighted number (30). The simple mode method provides consistent estimates of causal effects if at least 50% of SNPs are valid (31).

#### 2.3.3 Sensitivity and heterogeneity analysis

A leave-one-out sensitivity analysis was performed to examine the effect of individual SNPs on causal estimates. The examination of heterogeneity involved the utilization of Cochran’s Q statistic and the related *P-*values to ascertain the consistency of causal relationships across all SNPs. The consideration of smaller heterogeneity is deemed indicative of more reliable MR estimates, affirming the robustness and reliability of the causal inferences drawn from the analysis.

#### 2.3.4 MR Pleiotropy Residual Sum and Outlier (MR-PRESSO) analysis

The MR-PRESSO analysis was employed to assess the pleiotropy effects of outlier SNPs and correct abnormal findings attributable to such outliers (32). This method involves regressing SNP outcomes on SNP exposure and utilizing the square of residuals to identify outliers. Firstly, the MR-PRESSO global test was utilized to determine heterogeneity and outliers. Then, the MR-PRESSO outlier test was used to correct for pleiotropy by eliminating outlier SNPs. Ultimately, the MR-PRESSO distortion test analyzed the causality difference before and after outlier SNP removal (32). All the analyses were performed utilizing the R “TwoSampleMR” (v0.5.7, Stephen Burgess, Chicago, IL, USA) for the 2SMR analysis between T2DM and male infertility.

## 3 Results

### 3.1 MR analysis

To investigate the role of T2DM in the risk of male infertility, 2SMR methods were employed to identify relevant genetic variants in this study. 62 SNPs among European ancestry participants were associated with T2DM at the significance level of *P <*5×10^-8^. Then 62 independent SNPs (*F >*10) surpassed the limited value (*r* ^2^ <0.001) in LD analysis. Detailed information on the same of SNPs is also summarized **Table 1**.

**Figure 2:**
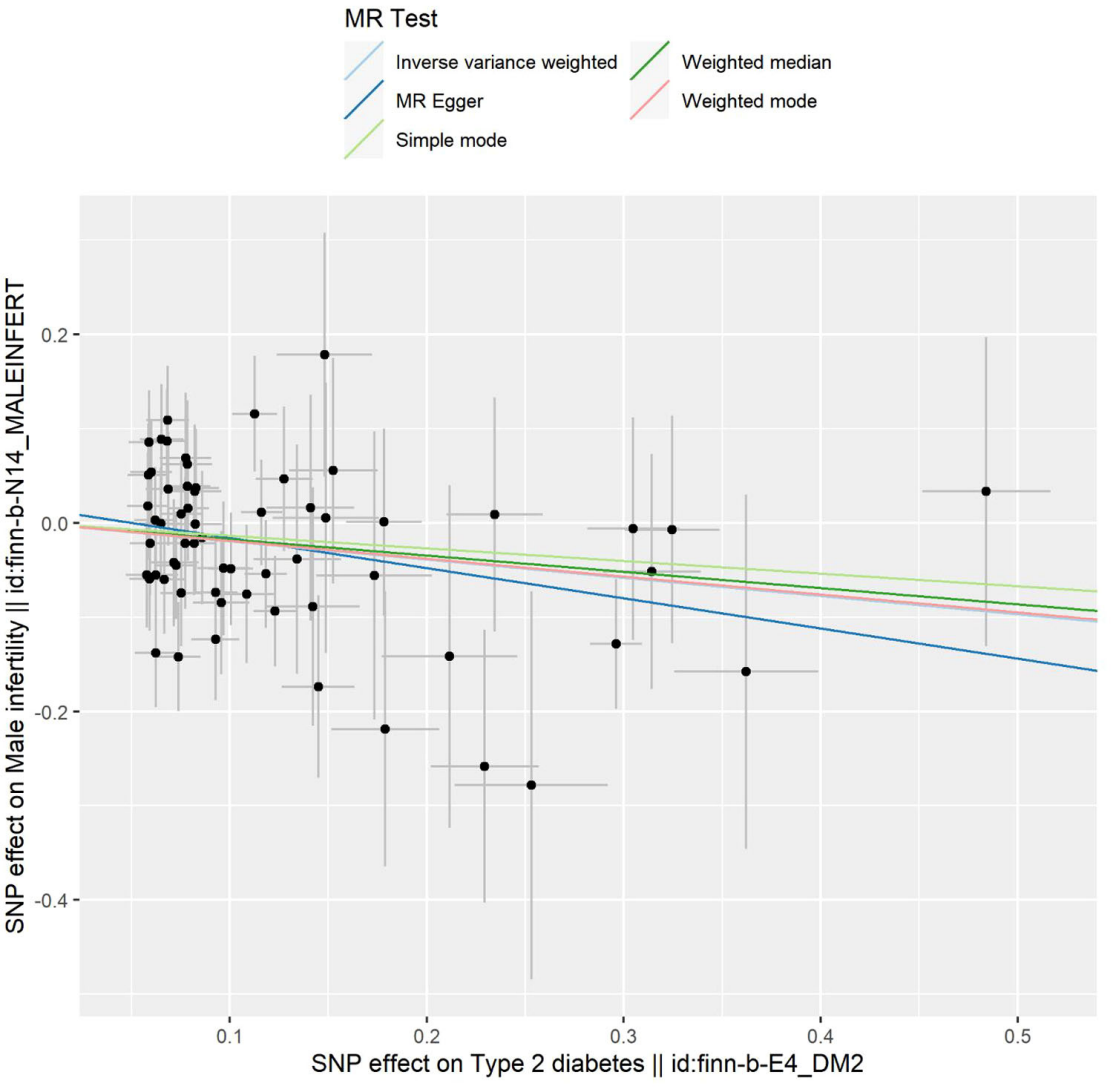
Scatter plot depicting the distribution of individual ratio estimates of type 2 diabetes mellitus with male infertility as the outcome. Trend lines generated from five different 2SMR methods are incorporated in all scatter plots to depict cause and effect.

The outcomes of the five 2SMR methods employed in this study are detailed in **Table 2** and illustrated in **Figure 2**. Notably, the IVW method served as the primary approach for estimating the causal effects of T2DM on the risk of male infertility. The study results revealed a substantial causal association between T2DM and the risk of male infertility in the European population (IVW fixed effects method: OR 0.824, 95% CI 0.703-0.966; *P*=0.017) (**Table 2** and **Figure 2**).

**Table 2:** Associations between genetically predicted T2DM and risk of male infertility.

Methods	OR	95%CI of OR	P value
MR Egger	0.726	0.527-1.001	0.056
Weighted median	0.841	0.654-1.082	0.178
Inverse variance weighted (multiplicative random effects)	0.824	0.700-0.970	0.020
Simple mode	0.875	0.544-1.405	0.581
Weighted mode	0.827	0.596-1.150	0.263
Inverse variance weighted (fixed effects)	0.824	0.703-0.966	0.017

OR, odds ratio; CI, confidence interval.

### 3.2 Sensitivity and heterogeneity analysis

Sensitivity analysis was executed to validate the reliability of the outcomes obtained from the IVW method. The findings of the IVW and MR-Egger test for heterogeneity analysis indicated the absence of statistically significant heterogeneity between T2DM and male infertility in all five 2SMR analysis methods (*P >*0.05) (**Table 3**).

**Table 3:** Sensitivity and heterogeneity statistics of two-sample Mendelian randomization analysis.

Method	Q value	Degrees of freedom	p-value
MR Egger	62.792	60	0.378
Inverse variance weighted	63.631	61	0.384

### 3.3 Further validation of MR results

**Figure 3:**
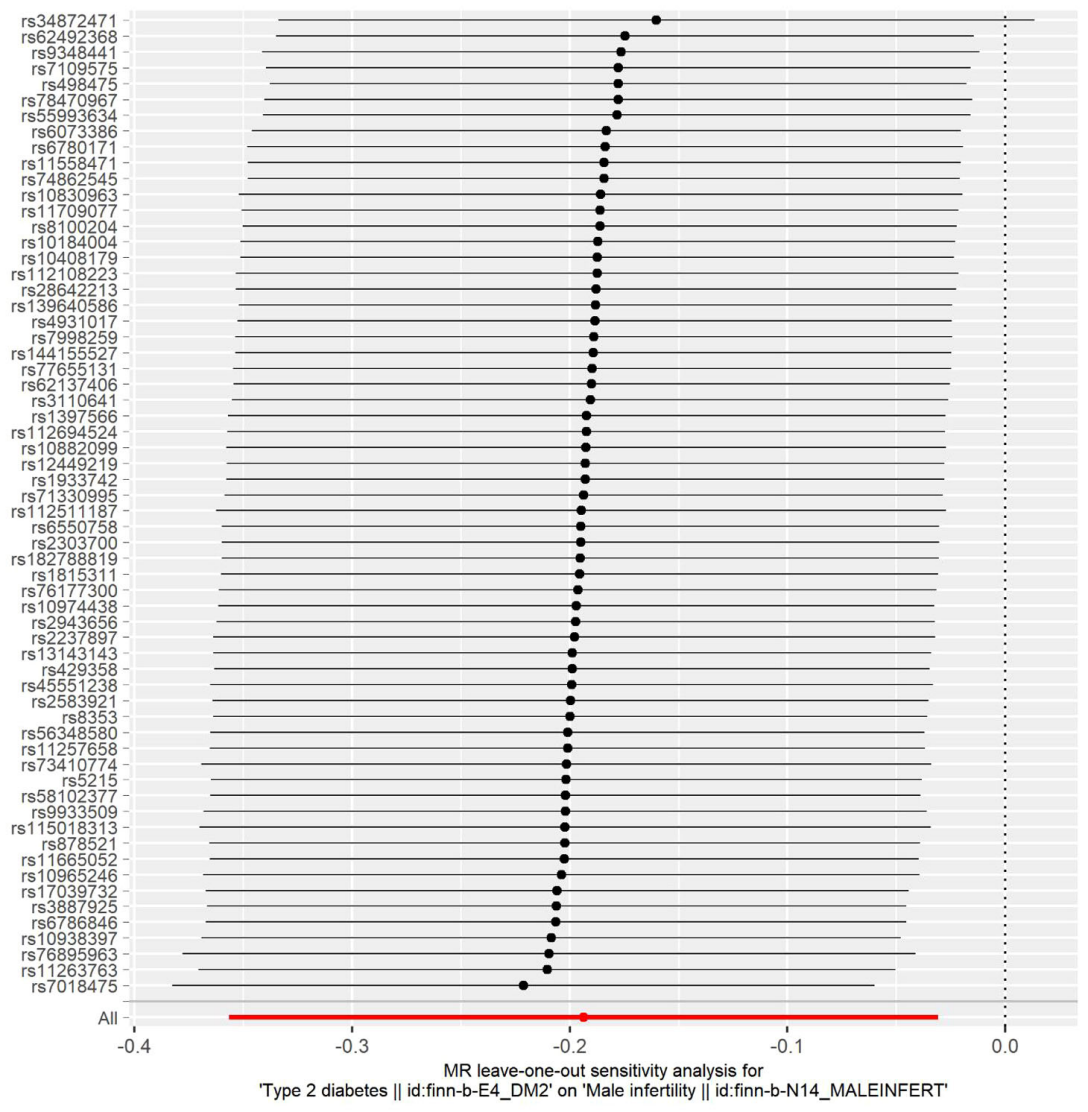
Leave-one-out sensitivity analysis for type 2 diabetes mellitus on male infertility. The dark dots in the visualization represent effect measures derived through IVW-MR analysis, with the exclusion of specific SNPs. Red lines denote the pooled analysis, incorporating all SNPs through the IVW-MR method, and are plotted for the purpose of comparison.

In this study, the selection of SNPs adhered to the genome-wide significance level criterion of *P <*5×10^–8^, aligning with the first condition—the locus-wide significance threshold. As demonstrated by the leave-one-out analysis (**Figure 3**), individual SNPs were observed to potentially influence the results of the IVW analysis. Consequently, further verification of the IVW method results was undertaken. Furthermore, the results of the MR-Egger regression intercept analysis did not reveal any substantial directional horizontal pleiotropy (*P >*0.05) (**Table 4**). The MR-PRESSO analysis verified the absence of marked horizontal pleiotropy and outliers in this research(*P*>0.05) (**Table 5**), further affirming the validity of the MR findings. Overall, the outcomes of this research demonstrate a substantial causal relationship between T2DM and the risk of male infertility in the European population.

**Table 4:** Pleiotropy statistics of two-sample Mendelian randomization analysis.

Method	Egger regression intercept	Standard error	Directionality p-value
MR-egger	0.015935	0.01779	0.3740

**Table 5:** Pleiotropy and outliers statistics of MR-PRESSO analysis.

	Exposure	MR Analysis	Casual Estimate	Standard Deviation	T-statistics	p-value
Main-MR	T2DM	Raw	-0.194	0.0831	-2.33	0.0231
	T2DM	Outlier-corrected	NA	NA	NA	NA
RSSobs of Global Test in MR-PRESSO result				65.569		
P value of Global Test in MR-PRESSO result				0.363		

NA, Not applicable.

## 4 Discussion

In the present investigation, a 2SMR analysis was conducted using publicly available GWAS summary statistics data. The objective was to examine the causal relationship between T2DM and male infertility in the European population. As per the currently available literature, the present research appears to represent the initial attempt to examine and unveil a causal relationship between genetically predicted T2DM and the risk of male infertility in the European population. This contribution is deemed significant in providing insights into the mechanisms underlying the association between T2DM and male infertility.

DM can inflict permanent damage on multiple physiological systems and various organs, inclusive of the reproductive organs, potentially resulting in dysfunction or failure of these systems (33). The mechanism of diabetic testicular tissue damage includes glucose and lipid metabolism, oxidative stress, inflammatory response, endoplasmic reticulum stress, autophagy and so on. Eventually, the inflammatory infiltration of the testicular cells, the number of sperm, the decrease of energy, the obstruction of ejaculation, the decrease of male fertility. Presently, the rapid increase in the incidence of T2DM among adolescents, particularly males, is anticipated to significantly increase the prevalence of reproductive dysfunctions in males (34). In a retrospective analysis study, a 51% prevalence of subfertility was identified among individuals diagnosed with T2DM (35). It was reported that around 1.2% of infertile males had T2DM among a cohort of over 500 male partners of infertile couples (36). The prevalence of infertility in the male population with T2DM reached 35.1%, representing a significant increase compared to the normal population (37).

DM patients had higher risk of becoming male infertility and the mechanisms of damage reproduction were different in T1DM and T2DM (10). T1DM caused low ejaculate volume and mitochondrial damage resulting in decreased sperm motility. T2DM caused an inflammatory condition with increased oxidative stress, resulting in decreased sperm vitality and increased sperm DNA fragmentation (10). In addition, various inflammatory signaling pathways and cell growth signaling molecular mechanisms also affect the proliferation, differentiation and death of testicular cells. T2DM may lead to dysregulated spermatogenesis, impairment of erectile function and ejaculation disorders, thereby impairing male fertility (38, 39). Furthermore, numerous studies have shown that DM affects male fertilization by inducing reactive oxygen species (ROS), which negatively affects sperm development (40–42). However, the role of uncoupling proteins (UCPs) as key regulators of redox homeostasis and ROS production in the pathophysiology of diabesity, as well as their potential involvement in diabesity-induced male infertility, remains a subject of debate (43). In addition, the treatment of rats in T2DM restored steroidogenesis in their testes, leading to improve spermatogenesis (44). Other studies also showed that treatment of T2DM increased sperm survival in pigs and improved the quality of frozen sperm in dogs (45). However, *in vivo* studies from a variety of animal models were found that inconsistent effects of T2DM treatment on sperm count, concentration, morphology, viability and survival (46). The exact molecular mechanism of male infertility in diabetes is unknown, and no specific drug is available to treat it.

The acknowledgment of potential confounding factors in previous studies prompted our approach to utilize MR analysis in this study. Recognizing the primary advantage of MR in removing the impact of confounding factors, this research aimed to enhance the reliability of the outcomes (47). Nevertheless, the findings from an MR study indicated that the relatively elevated risk of abnormal spermatozoa in the European population cannot be solely explained by T2DM (16). Given the inconsistent findings observed in previous studies, it becomes imperative to conduct subsequent investigations with larger sample sizes to elucidate the relationship between T2DM and male infertility. Consequently, this research structured a 2SMR study to unveil the causal relationship between T2DM and the risk of male infertility in the European population. Multiple studies conducted in animals and humans have consistently highlighted the adverse effects of DM on male reproductive functions (34). The present research were consistent with previous research.

Nonetheless, the findings of the present research contrast the MR study (16) that analyzed the causal relationship between T2DM and abnormal spermatozoa in the European population. The differences between the two analyses arise from distinct exposures and outcomes, variations in data sources, and differences in the number of cases and SNPs considered. Firstly, the analyses focused on different outcomes, contributing to the divergent results. Secondly, dissimilar datasets were utilized in the two analyses. Thirdly, there was a discrepancy in the number of cases and SNPs between the studies. The prior analysis identified 17 SNPs associated with T2DM, and 9 independent SNPs related to the abnormal spermatozoa surpassed the limited value in LD analysis (16). However, this study identified 62 SNPs associated with T2DM, and among them, 62 independent SNPs related to male infertility surpassed the limited value. In general, the outcomes of the current research align with numerous studies analyzing the effects of T2DM.

This study possesses several strengths. The implementation of a 2SMR analysis provided a robust framework for the investigation of the causal association between T2DM and male infertility. This methodological approach served to exclude the interference of confounding factors and reverse causation on causal inference, thereby enhancing the validity of causal inferences. Moreover, the genetic variants linked to T2DM were sourced from the most extensive and comprehensive GWAS summary data available, ensuring the robustness and strength of the instruments employed in the MR analysis. Moreover, in this study, a thorough examination was conducted through leave-one-out sensitivity analysis and heterogeneity analysis. Horizontal pleiotropy, a potential confounding factor, was identified and addressed utilizing MR-PRESSO and MR-Egger regression intercept analyses. It is noteworthy that no evidence of horizontal pleiotropy was noted in this study. The adoption of a 2SMR approach, coupled with the utilization of nonoverlapping exposure and outcome summary-level data, was a strategic measure employed to avoid bias and enhance the overall robustness of the findings of the study (48).

Nevertheless, it is crucial to acknowledge various limitations in this research when interpreting the outcomes. Firstly, the study participants are exclusively of European ancestry, limiting the generalizability of the findings to the broader population. Secondly, potential confounding factors, including age, gender, and environmental influences, may introduce variability in the MR analysis. Furthermore, this research exclusively determines the causal associations between T2DM as the exposure and male infertility as the outcome. Future research will explore the reverse causal associations, examining male infertility as the exposure and T2DM as the outcome.

To conclude, this research offers novel insights into the genetic basis of the causal relationship between T2DM and male infertility, offering valuable guidance for future research endeavors.

## Data availability statement

The original contributions presented in the study are included in the article/supplementary material. Further inquiries can be directed to the corresponding author.

## Ethics statement

Ethical approval was not required for the study involving humans in accordance with the local legislation and institutional requirements. Written informed consent to participate in this study was not required from the participants or the participants’ legal guardians/next of kin in accordance with the national legislation and the institutional requirements.

## Author contributions

CF: Conceptualization, Data curation, Formal Analysis, Funding acquisition, Investigation, Methodology, Project administration, Writing – original draft. JZ: Data curation, Formal Analysis, Investigation, Methodology, Project administration, Software, Supervision, Validation, Visualization, Writing – review & editing. DQ: Investigation, Supervision, Validation, Writing – review & editing.
